# Research on the Direction of Innovation and Entrepreneurship Education Reform Within the Digital Media Art Design Major in the Digital Economy

**DOI:** 10.3389/fpsyg.2021.719754

**Published:** 2021-10-15

**Authors:** Wenhui Yu, Tan Jiang

**Affiliations:** ^1^Department of Art and Design, Shanghai Publishing and Printing College, Shanghai, China; ^2^School of Fine Art, South China Normal University, Guangzhou, China

**Keywords:** innovation, entrepreneurship, digital media art design, professional education, talent training

## Abstract

This research analysed the integration of innovation and entrepreneurship education for students, majoring in digital media art design. Using a grounded theory approach, we investigated the experiences of two research groups: five leaders of digital media art design courses in colleges and universities, and five successful entrepreneurs who had majored in digital media art design and had achieved significantly in the industry after graduation. From the two dimensions of ‘innovation’ and ‘entrepreneurship’, as well as the two perspectives of ‘implementer’ and ‘party’, semi-structured interviews were conducted on the core competencies that innovation and entrepreneurship talents should have the methods and experience of innovation and entrepreneurship education, the difficulties and problems in the implementation of innovation and entrepreneurship education, the need for talent among enterprises and the factors affecting entrepreneurship success. By analysing and clustering the data, we were able to comprehensively identify the main problems and aspects that require more attention in terms of cultivating innovative and entrepreneurial talents in the digital media art design major. Based on the grounded theory research method, this paper established a theoretical model, outlining the innovation of the digital media art design major and the entrepreneurship education reform path. The key internal elements of the model include mechanisms for professional talent training, curriculum integration, teacher team construction and resource support. The school-enterprise cooperation mechanism is recognised as the key external element for innovation and entrepreneurship education reform. The results provide direction for future education and teaching reform, as well as professional input for the digital media art design major. The findings encourage those involved in the digital media art design major to cultivate more high-level, pioneering professionals, so as to adapt to the transformation and upgrading of economic and social development in the context of a growing digital economy.

## Introduction

### Research Background

Digital media has become a core industry and a new area of economic growth, with great vitality and potential in the 21st century knowledge economy. Against the background of a rapidly developing digital economy that promotes digital industrialisation, industrial digitization and digital governance, the digital media industry effectively combines human creativity and technology. At the same time, the rapid development of the digital economy and information technology has promoted the rise of global entrepreneurship. Studies show that entrepreneurial activities play an important role in scientific and technological progress, economic development, job creation and employment structure improvement (Fan et al., 2020). In China, the ‘mass entrepreneurship and innovation’ policy has become an important part of the national employment macro policy guidance. According to the 2016–2017 Global Entrepreneurship Observation Report, China’s entrepreneurial activities are effective, due to innovative global economic development and a drive towards innovation efficiency (Wu, 2015).

The rapid development of the digital economy has promoted the transformation and upgrading of global economic and social development, and driven profound changes in the education ecosystem. In recent years, with the government’s attention focused on innovative industries and policy support for entrepreneurial activities, the entrepreneurial development of innovative industries has increased. However, the innovation and entrepreneurial development of the digital media industry are still unable to keep pace with national economic development. Digital transformation and the upgrading of the current economy have led to an adjustment of the industrial structure and the implementation of an innovation-driven development strategy. Standards for the social consideration of technical and skilled talent are no longer limited to practicality, operability or other single indicators of skills, but tend to be diverse, with a focus on comprehensive professional qualities and sustainable development abilities. Innovative entrepreneurship – together with professional and technical talent – will become an important development trend of the future economy and society.

In 2002, the Ministry of Education in China started a pilot of entrepreneurship education with nine universities, gradually promoting and implementing it in the national universities. With the continuous advancement of entrepreneurship education reform policy, many colleges and universities paid more attention to the cultivation of innovative and entrepreneurial talents, promoting innovation and entrepreneurship education, with a focus on public opinion guidance, policy guarantee and financial support. These aspects have achieved remarkable results in promoting students’ entrepreneurial awareness and project exploration (Liu and Liu, 2017). However, data show that of the approximately 7million college graduates in China every year, only around 2% are independent entrepreneurs, and the entrepreneurial success rate of this group is generally low (Wu, 2015). According to the ‘employment report of Chinese college students in 2020’, the proportion of self-employment of graduates in China in 2019 was approximately 2.5%, essentially the same as in 2018 (2.7%) and 2017 (2.9%; Lu et al., 2021).

Most application-oriented colleges and universities have not implemented entrepreneurship education as part of talent training and have no plans to include it in their professional talent training programmes (Zhao, 2014). The strategies adopted by colleges and universities in innovation and entrepreneurship education mainly focus on setting up innovation and entrepreneurship education courses, guiding students to participate in science, technology and entrepreneurship competitions, carrying out entrepreneurship knowledge lectures and providing basic information. These methods are minimally related to the real entrepreneurship activities conducted by professional innovators.

### Research Purpose and Significance

Innovation and entrepreneurship education are key themes of the digital age. Combining professional education with innovation and entrepreneurship education is a practical challenge in relation to developing education in the new era. Digital media design is a new comprehensive discipline across natural sciences, social sciences and humanities. Urgent thought and exploration are needed to implement vocational education, strengthen the cultivation of high quality, technical and applied digital talents to develop a smart society and to integrate innovation education and entrepreneurship education into professional education. This integration is needed for digital media professionals to meet the demands of a rapidly developing digital economy (Yu et al., 2021). By interviewing the leaders of digital media art design courses in sample colleges, as well as successful entrepreneurs, who majored in digital media art design and are making significant achievements after graduation, this study describes and analyses the research content from the two dimensions of ‘innovation’ and ‘entrepreneurship’ and the two perspectives of ‘implementers’ and ‘parties’. The study has also obtained more comprehensive analysis materials than previous studies and uses the grounded theory research method to encode and analyse the original interview materials step-by-step. This enables the comprehensive identification of the main problems and aspects that require more attention in the cultivation of innovative and entrepreneurial talents in the digital media art design major, and an analysis and summary of the main problems and their correlation structure in the cultivation of students’ innovative and entrepreneurial ability in digital media art design at higher education level. This study also helps establish the theoretical model of the innovation and entrepreneurship education reform path of the digital media art design specialty and finally, establishes the innovation and entrepreneurship education reform path model, suitable for the digital media art design specialty. The research results provide a directional reference for the structure of education and teaching reform within the digital media art design specialty.

### Literature Review

Entrepreneurship education originated from economics, focusing on the entrepreneurial knowledge required by business schools to encourage learners to start small businesses (Liu et al., 2014). The rise of the entrepreneurial economy has created more demand for entrepreneurship education, expanding from business schools to other settings. After more than 60years of development, countries like the United States and the United Kingdom have formed a sound entrepreneurship education system (Li et al., 2013). Our literature search revealed that foreign scholars devote considerable thought to the research of entrepreneurship education methods, and many of the approaches are premised on the basic concepts and methods of entrepreneurship education. Fiet discussed the teaching strategy of entrepreneurship education courses and maintained that teachers should establish a classroom system in which students can improve their ability through theory-based practice (Fiet, 2001; Chen et al., 2021). Similarly, Rasmussen et al. consider that entrepreneurship education should be action-oriented – emphasising learning by doing – and they put forward suggestions on establishing action-oriented entrepreneurship education (Rasmussen and Sorheim, 2006). Edelman et al. found differences between the actual activities of start-ups and the cases in entrepreneurship textbooks and suggested that the curriculum content of entrepreneurship education should be updated (Edelman et al., 2008).

Many scholars have undertaken comparative analysis of the research ‘hotspots’ of entrepreneurship education on the global higher education stage and have put forward relevant suggestions for innovation and entrepreneurship education in Chinese universities. Liu et al. used CiteSpace visualisation software to compare and analyse the research hotspots of entrepreneurship education locally and internationally from 1998 to 2017. The study found certain high-frequency keyword nodes in the knowledge map of domestic entrepreneurship education research, including ‘entrepreneurship education’, ‘innovation and entrepreneurship education’, ‘college students’, ‘colleges and universities’, ‘higher education’, ‘entrepreneurship education’ and ‘entrepreneurship’. Based on this, it is inferred that domestic entrepreneurship education research mainly focuses on entrepreneurship education research in colleges and universities (Liu and Zhang, 2020). CiteSpace visualisation software was also used by Liu et al. to compare the research status, hot spots and development trends of 667 CSSCI papers and 685 SSCI papers (Liu et al., 2018; Chen et al., 2019). They found that research into the field of innovation and entrepreneurship education in China remains focused on the theoretical connotations of entrepreneurship education, studying the influence of education quality, professional education and other educational reform methods on talent cultivation and the university employment rate. Compared with the micro focus of international research, Chinese research tends to explore more macro concepts. The present study emphasises the future research direction for innovation and entrepreneurship education in China, which should transition from a macro approach to more substantive, detailed research, that explores the influencing factors and specific paths of entrepreneurial behaviour, based on theoretical knowledge.

In terms of innovation and entrepreneurship education reform strategies, certain scholars have put forward macro strategic suggestions, based on the analysis of their own research fields and the characteristics of innovation and entrepreneurship education in China. Li et al. proposed building a national cloud entrepreneurship platform for college students, integrating resources to effectively form an industrial chain to open a new mode of entrepreneurship education (Li et al., 2012; Chen et al., 2019b). Yao et al. proposed that entrepreneurship education could comprehensively optimise college students’ entrepreneurial practice quality through progressive, embedded and competitive modes (Yao et al., 2016). Based on experiential learning theory and from the perspective of the Communist Youth League (the main body of experiential education), Zhao et al. constructed a ‘five in one’ entrepreneurship education mode with the popularisation of entrepreneurship knowledge as its premise (Zhao and Fang, 2015). Entrepreneurship observation experience was used as a guide, and an entrepreneurship planning competition, simulating entrepreneurship, was used as the platform. Hu et al. suggested domestic innovation and entrepreneurship education, such as cultivating campus innovation and a culture of entrepreneurship (Hu and Shen, 2013). They suggested constructing a multi-level organisational structure of innovation and entrepreneurship education through the introduction and comparative analysis of the renowned entrepreneurship curriculum system reform of the Baisen Business School and the entrepreneurship education mode of Stanford University. Hu et al. put forward an ‘entrepreneurship centre model’ – a mix of typical entrepreneurship education organisation models in China and the United States – suitable for entrepreneurship education in colleges and universities in China (Hu and Chang, 2016; Chen et al., 2019b).

Scholars have carried out a preliminary analysis of the factors influencing innovation and entrepreneurship education in China. Guo pointed out that in developing the innovation and entrepreneurship curriculum in China, a hierarchical structure is logical, but the curriculum model is a relatively simple one with a unitary focus (Huang et al., 2019; Guo, 2021). The curriculum is diverse but very theoretical. The curriculum system is relatively comprehensive, but its cohesion needs to be improved. Furthermore, the legitimacy mechanism for professional degrees needs to be improved. Qiu considers that the cultivation of innovation and entrepreneurship teaching in colleges and universities is still in the primary stage (Chen et al., 2015; Qiu, 2021). Innovation and entrepreneurship teachers lack entrepreneurial experience. Most are part-time teachers, and their practical teaching methods are divorced from actual, entrepreneurial behaviour. Zhong studied the external factors affecting innovation and entrepreneurship education by establishing and analysing the five-circle model (Huang et al., 2019; Zhong, 2021). Optimization of the innovation and entrepreneurship environment of application-oriented universities can be described from the perspective of ideological understanding, policy provisions and systems and mechanisms, based on the relevant theories of education and management, and the three perspectives of government, university and society (Yang, 2020).

Within the aspect of innovation and entrepreneurship education of the digital media art design specialty, certain scholars have also expounded and explored the training mode of innovation and the entrepreneurship talents of the digital media specialty, the fostering of students’ innovation and the entrepreneurship training plan system, the establishment of an innovation and entrepreneurship curriculum system, the creation of an entrepreneurship centre and management strategies and the importance of an innovation and entrepreneurship curriculum system within the digital media art specialty. Hu investigated three specific modes of innovation and entrepreneurship education practice in domestic colleges and universities and pointed out that, due to professional differences, the three modes of innovation and entrepreneurship practice have limited relevance for digital media art and design education (Hu, 2015). Ding analysed the integration of innovation and entrepreneurship education, as well as professional education, and elaborated on the ‘embedded mode’ for integrating innovation and entrepreneurship education and professional education (Huang et al., 2015; Ding, 2018). A series of practices for integrating innovation and entrepreneurship ability training into professional teaching practice was summarised by Wang for his university (Huang et al., 2012; Wang, 2019). He pointed out that innovation and entrepreneurship education should run through the whole process of higher education training through multi-party cooperation and joint participation. Guo emphasised that the innovation and entrepreneurship training programme for college students majoring in digital media has the characteristics of the combination of technology and art, the combination of concept and practice and the combination of innovation and application. This is the first time that the idea and reform direction of cultivating students’ scientific spirit and scientific research consciousness have been put forward, as well as the notion of developing college students’ innovative thinking and innovative ability, as a result of students participating in teachers’ scientific research activities (Guo et al., 2016; Huang et al., 2017). At the same time, for example, Chen Kun, Li Aimin, Shu Mei and other scholars have studied and expounded certain local, practical cases, based on their own problems and experiences in the implementation of innovation and entrepreneurship education within the digital media art design specialty in colleges and universities.

Through literature research, it has been found that, at present, within the research content of the relevant research fields of this subject, more research results reveal the current situation of innovation and entrepreneurship education, and most of the research on the reform direction of innovation and entrepreneurship education are focused at macro policy level; there are few discussions on how to organically combine innovation and entrepreneurship education with professional talent training. In terms of the research perspective, based on the outlook of the department of digital media art and design, there are few targeted research results focusing on the characteristics of digital media art and design; the research method is also rather limited and few research results focus on the characteristics of the digital media art and design specialty. Although the discussion and analysis of certain scholars have involved curriculum construction, teaching management and other aspects, this process is primarily managed in terms of summarising practical experience, especially the excavation and cause thinking of common problems in the specific implementation process, the correlation analysis between innovation and entrepreneurship education and the various influencing factors of digital media art design education; this aspect needs to be studied further.

This study focuses on digital media art design, an applied major that focuses on creativity and creative thinking in professional quality training, with students demonstrating a considerable willingness and demand for entrepreneurship. Through interview and investigation, based on the research method of grounded theory, this paper discusses the main problems and aspects that require more attention in cultivating innovative and entrepreneurial talents in the digital media art design specialty. The study also considers the correlation between the influencing factors of the digital media art design specialty, as well as innovative and entrepreneurial education, so as to establish a theoretical model of the reform path of innovative and entrepreneurial education within the digital media art design specialty. It provides a directional reference for education and teaching reform and the professional establishment of the digital media art design specialty.

## Research Design

### Research Approach

Digital media is an interdisciplinary subject, integrating technology and art. It is based predominantly on information technology and the theory of mass communication. It covers a broad development space and has great potential for further growth, involving knowledge of plastic arts, art design, interactive design, computer software technology, graphics and image processing, and information and communication technology. As part of the media industry, it mainly cultivates students’ artistic talents and digital product art design. This major requires students to be skilled in traditional art modelling and design and to have a mathematical foundation. It is necessary to think both imaginatively and in a strictly linear, logical way, which coincides with innovation and entrepreneurship education concepts (Bonnie and Welsh, 2021).

Based on the actual development of innovation and entrepreneurship education in colleges and universities, this study takes the digital media art design major as an example to explore the innovation and entrepreneurship training mechanism of application-oriented professionals. We attempt to build a model of innovation and entrepreneurship training for this major to provide direction for innovation and entrepreneurship teaching reform in the field of digital media art design. The study findings may also provide a reference for other applied majors that focus on cultivating creativity.

### Research Method

Grounded theory is the main approach used in this research. Grounded theory research is a qualitative research method, which establishes substantive theory from the bottom up. It is a logical process of collecting and analysing data, and forming theories, created by Anselm Strauss and Barney Glaser of Columbia University in the United States. The primary purpose of the theory is to analyse and deduce the core concepts or ‘the essence’ from empirical data. Initially, there is no theoretical hypothesis. Researchers start with the initial text data for mining and analysis, obtaining certain initial concepts and empirical generalisations, and then integrating these initial concepts, considering the relationship between them in more detail and connecting them. Finally, further analysis of the relationship is undertaken by mining integration and constructing a theory with practical significance (Glaser et al., 1968). As a method of germplasm research, grounded theory was first applied to sociological research and then gradually spread to many fields, such as pedagogy, psychology and management. After continuous improvement and improvement by scholars at home and abroad, it has been widely recognised as an effective way of building localization with theory. This exploratory research process is very consistent with the direction of this research.

On the other hand, the innovation and entrepreneurship education system in colleges and universities do not exist in isolation, especially in the case of the applied specialty of digital media art design, which focuses on cultivating creativity and creative thinking. The cultivation of professional ability and innovation, as well as entrepreneurship ability, is strongly related in terms of methods and training objectives. The standardised, qualitative data analysis method, based on grounded theory, can accurately analyse various factors related to innovation and entrepreneurship education and the professional education of the digital media art design specialty through data research (Song et al., 2011). This method is also able to correctly handle the relationship between various influencing factors and grasp the problem of dynamic balance between them, providing a strong guarantee for the effectiveness and credibility of the reform path of innovation and entrepreneurship education, suitable for the digital media art design specialty.

### Research Object

The selection of research objects is based on the following principles: firstly, select the purposeful sampling method, according to the characteristics of grounded theory research methods; secondly, qualitative research needs to select samples to understand the research problems.

In terms of selecting research subjects, we chose interviewees from the two dimensions of innovation and entrepreneurship. Teachers in universities were used to represent innovation, with five professional leaders of the major in digital media art design in colleges and universities being invited to participate. These professional leaders are teachers, carrying out both teaching and research in the digital media field. Semi-structured interviews were used to obtain information on the core competence of innovation and entrepreneurship talents, the nature and experience of innovation education and the difficulties in implementing innovation and entrepreneurship education.

The representatives of entrepreneurship education were successful entrepreneurs – five entrepreneurs who had majored in digital media art design in higher education and had achieved significantly in the industry after graduation. The entrepreneurs had worked in the industry for more than 3 years and had made significant achievements. Semi-structured interviews were used, mainly focusing on their own experience, enterprise needs, talent recruitment and entrepreneurship success factors. Through the two dimensions of ‘innovation’ and ‘entrepreneurship’ and the two perspectives of ‘implementers’ and ‘parties’, this paper provides an in-depth and comprehensive description and analysis of the research content and obtains more rich and practical research materials.

### Data Collection and Sorting

This study uses the method of semi-structured interviews to obtain the original analysis material information. Since the interviewees were selected from different universities and companies, the research conducted one-to-one in-depth interviews on the relevant contents of the research scope by means of offline, face-to-face interviews. Focusing on the theme of innovation and entrepreneurship of digital media art design, an interview outline and discussion direction, with different emphases, were designed in the interview process, according to the identity of the respondents. With the permission of the interviewer, the interview content was recorded to ensure the integrity of the collected data.

### Methodology and Research Process

This study followed the conventional analysis process of grounded theory and analysed the interview text data in a step-by-step, coding process. To obtain the main modules of innovation and entrepreneurship education reform of the digital media art design major, we sorted through the relevant data and materials, eliminated irrelevant information and carried out layer-by-layer coding, using open coding, principal axis coding and selective coding. We then constructed a theoretical model to account for the innovation and entrepreneurship education reform of the digital media art design major.

#### Open Coding

Loose coding is the process of conceptualising and categorising the original data, follow the process of ‘original data - Labelling - Conceptualization – Categorization’ to sort out the original data. Data are broken up, summarised, sorted and refined. Concepts are formed and categories related to the concept are found, finally focusing on the problem (Liu et al., 2016). Through data analysis at this stage, 43 initial concepts, related to innovation and entrepreneurship education in digital media art design, were formed. These can be summarised into 17 categories. Table 1 shows the coding of selected initial concepts and categories.

**Table 1:** Examples of open coding.

Category	Initial Concept	Interview Data Direct Quotations from Participants
A1 The resources and content of the innovation and entrepreneurship courses are incomplete	a1 Curriculum resources are also very scarce and there is no independent and systematic entrepreneurship curriculum group	There is only one compulsory course related to innovation and entrepreneurship, and it is a theoretical course.
		There are no elective courses related to entrepreneurial ability.
		Students have a very limited choice of entrepreneurship courses.
		The innovation and entrepreneurship course is run by the student affairs department, which does not belong to the college management.
		In addition to the independent, extracurricular innovation and entrepreneurship, this is mainly implemented through the ‘second classroom’ scientific and technological training, subject competition and entrepreneurship and entrepreneurship lectures, as well as the entrepreneurship training, practical training and entrepreneurship practice that a small number of students participated in.
	a2 The content of the innovation and entrepreneurship course is based on general theory, and the structuring of the course is still in the exploratory stage	The course does not mention certain preferential policies for entrepreneurship, provided by national or regional governments, and there is no information on entrepreneurship financing.
		The enterprise operation mode, organisational structure, human resources and other aspects of the course are not included.
		Entrepreneurship is a complex and interdisciplinary field of knowledge, which involves not only entrepreneurial knowledge, but also teamwork and psychological quality.
		Most of the cases in the innovation and entrepreneurship course content concern the introduction of achievements, while the introduction of processes is more limited.
		To start a business immediately after graduating, it is vital to know a great deal about enterprise operation, however, it is really difficult to cover this in a course of 30h.
		There is a considerable difference between the content learned in class and the actual situation, and the practical content is also limited.
	a3 The cases in the innovation and entrepreneurship course cannot cover all majors	There is a considerable difference between the content learned in class and the actual situation. There is too little practical content. Innovation and entrepreneurship courses are generally large courses. The whole year group is set up in a unified way. Corresponding adjustments, according to the characteristics of different majors and different students are not made, and it is not easy to attract students’ attention.
		Each specialty has its own characteristics and the operation modes of enterprises, with different directions in terms of experience are certainly different.
	a4 The innovation and entrepreneurship curriculum does not involve professional development trends and the major is not placed at the forefront	In fact, the innovation and entrepreneurship course is still a general course, which is essentially a large course across the entire school, so it does not specifically talk about the direction of professional development and other issues, but this information must be understood in relation to entrepreneurial project practice.
		Digital media major is an emerging industry. With the rapid development of technology, new directions in industry are constantly being refined. If students wish to start a business, they still need to understand this information.
		It is difficult to stimulate students’ entrepreneurial vitality if there is no practical link in the content of the innovation and entrepreneurship course.
		If students wish to start a business, they are likely to rely on their major.
		If students wish to start a business, they are likely to rely on their major. At that time, if the innovation and entrepreneurship course can include professional cutting-edge information and an introduction to the industrial market, it will make the course more focused.
	a5 The innovation and entrepreneurship course lacks the cultivation of innovation and entrepreneurship consciousness	The innovation and entrepreneurship course is a compulsory public course, and almost all the students of various majors have little interest in it.
		In fact, many students still believe that innovation and entrepreneurship have nothing to do with their major, their future development direction and career planning. They just come to class to complete credits.
		In fact, not all majors and all students are suited to entrepreneurship, but the cultivation of innovative thinking is needed by all majors in society now. Many students do not recognise this idea.
A4 The content of the innovation and entrepreneurship courses has limited relevance for professional courses	a13 The content of the innovation and entrepreneurship course does not cover the specific content of professional ability training	At present, entrepreneurship and innovation education is still a module independent of professional education, which is also included in the talent training programme, but is not integrated with professional education.
		When I was in school, the innovation and entrepreneurship class was just a theory class.
		I remembered that the practical element of the innovation and entrepreneurship class had nothing to do with my major.
	a14 The content of professional courses does not involve the cultivation of entrepreneurial ability	We put entrepreneurship education, discipline competition, entrepreneurship and innovation lectures as well as entrepreneurship practice in the category of ‘second classroom’, but the way in which entrepreneurship education is taught in the first classroom is given less consideration.
		The professional courses of the digital media art design major focus on the cultivation of innovation ability, but entrepreneurial ability is not considered.
		The cultivation of professional courses is universal, and not all students wish to start a business.
		The cultivation of professional courses is popular. Not all students wish to start a business. The main purpose of professional courses is to lay a good foundation of professional skills.
A12 Teachers have less experience in innovation and entrepreneurship	a36 Teachers of the entrepreneurship course are not professionals	The main purpose of professional courses is to lay a good foundation of professional skills. The teacher of the innovation and entrepreneurship course is not a professional teacher. He/she may be able to guide your creative project plan logically and structurally, but this is difficult to grasp if there is a deviation in relation to your entrepreneurial direction.
		The teacher of the innovation and entrepreneurship course is a counsellor, not a professional teacher.
		There are also certain schools in which innovation and entrepreneurship courses are taught by ideological and political teachers.
		Sometimes certain entrepreneurship lectures involve part-time teachers from enterprises.
	a37 Most professional teachers do not have direct, entrepreneurial experience or experience, and lack direct understanding and experience of entrepreneurship	Now there are many professional teachers who teach in colleges and universities as soon as they graduate, and they have no practical experience.
		School management does not encourage teachers to focus on off-campus projects or part-time jobs.
		Most of the teachers have a lot of transactional work within the school, and they are not able to spend time in enterprise.
	a38 There is a serious shortage of ‘dual qualified’ teachers who can engage in professional academic research and can integrate knowledge into entrepreneurial practice and research	There are few teachers with professional ability and entrepreneurial experience.
		Teachers with entrepreneurial ability and experience spend most of their energy in the company and are unlikely to spend extra time tutoring students.

#### Axial Coding

According to the related concepts, axial coding extracts more general concepts from the existing categories. Principal axis codes were used to classify, abstract and synthesise the most frequent and important concepts in the innovation and entrepreneurship education reform focus module of the digital media art design major. Categories were formed and further refined into principal axis categories to discover and establish connections between categories and concepts, and to distinguish between principal and subcategories. We conducted in-depth analysis of the principal axis category to grasp the context of the development of the event (Huo et al., 2019). According to the previous stage, the main problems in the cultivation of innovation and entrepreneurship education within the digital media art design specialty, obtained from the interview data, are reorganised. After combining the repeated conceptual factors, five main conceptual categories are summarised: curriculum accommodation mechanism, professional talent training mechanism, resource support mechanism, teacher team construction mechanism and school-enterprise cooperation mechanism. Table 2 presents a summary of the axial coding.

**Table 2:** Axial coding.

Main Category	Category
AA1 Curriculum integration mechanism	A1 The resources and content of the innovation and entrepreneurship course are incomplete
	A2 The teaching method of the innovation and entrepreneurship course is unitary
	A3 The practice of the innovation and entrepreneurship course is not rich
	A4 The content of innovation and entrepreneurship courses has low relevance for professional courses
AA2 Professional talent training mechanism	A5 The training to entrepreneurial quality in professional courses is not systematic
	A6 The training goal of professional education is relatively backward
	A7 The solidification of a professional talent training mode is required
	A8 The professional teaching management mode is not flexible enough
AA3 Resource support mechanism	A9 There is a lack of online project resource platforms
	A10 There is insufficient policy and financial support
	A11 There is a scarcity of entrepreneurial practice space and resources
AA4 Teacher team construction mechanism	A12 Teachers have limited experience in innovation and entrepreneurship
	A13 Few opportunities for teacher training and promotion
	A14 Teachers’ incentive policy is not clear
AA5 School-enterprise cooperation mechanism	A15 There is a limited level of school-enterprise cooperation
	A16 There is limited resource sharing between schools and enterprises
	A17 The school-enterprise feedback mechanism is not established

#### Selective Coding

**Figure 1:**
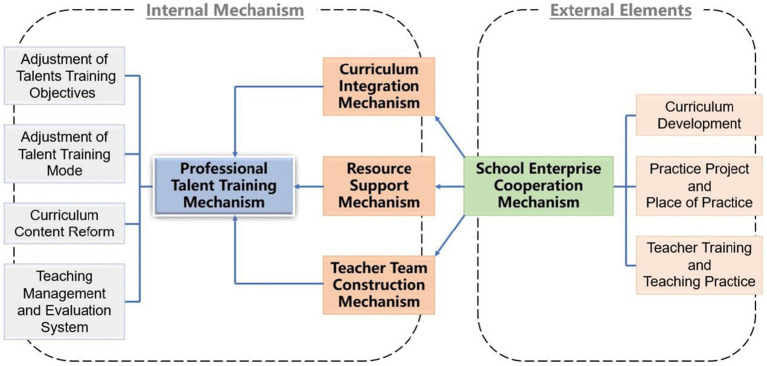
Theoretical model.

Selective coding is based on the core category, with the main category after analysis being connected to verify the relationship between categories. The core category is shown to be dominant in comparison with other categories so that the code, concept and category related to the core category can be included in a broader theoretical scope (Lu et al., 2018). The core purpose of this study was to use the grounded theory research paradigm to elucidate the main problem structure of innovation and entrepreneurship education in the digital media art design major. We set out to build a theoretical model of the education reform path for innovation and entrepreneurship within the digital media art design major, systematically connecting the core category with other categories, using a diagram as shown in Figure 1.

#### Model Interpretation

The theoretical model indicates five main mechanisms – professional personnel training, curriculum integration, teacher team building, resource support and school-enterprise cooperation – working together to build a reform path for professional innovation and entrepreneurship education within the scope of digital media art design. Establishing a professional talent training mechanism is the core, requiring the joint support of mechanisms for curriculum integration, teacher team building, resource support and school-enterprise cooperation. Of the four areas surrounding the core, three have an internal focus and one is linked to external aspects. The mechanisms for curriculum integration, teacher team construction and resource support are linked to the coordination and cooperation of the operation mechanism of colleges’ and universities’ internal systems. Together, these three aspects play a major role in establishing the innovation and entrepreneurship training system of the digital media art design major. The fourth element surrounding the core – school-enterprise cooperation mechanism – is also an indispensable element of the innovation and entrepreneurship ability of the digital media art design major. Although externally focused, this can assist the three internal areas of curriculum integration, teacher team construction and resource support from an external perspective.

### Research Results

Using the grounded theory research method, this study conducts a level-by-level coding analysis of the raw material of 10 interviews from the two concepts of ‘innovation’ and ‘entrepreneurship’ and the two perspectives of ‘implementer’ and ‘party’, and comprehensively identifies the main problems and aspects that require more attention in cultivating innovative and entrepreneurial talents in the major of digital media art design. By analysing and clustering the category and structure of the problems collected from the original data, the theoretical model of the innovation and entrepreneurship education reform path of the digital media art design specialty is established. Finally, the key internal elements, suitable for digital media art design, are the construction of the professional talent training mechanism, the curriculum integration mechanism, the teacher team construction mechanism and the resource support mechanism, with the school-enterprise cooperation mechanism acting as the external key element.

## Conclusion and Discussion

The digital media art design major is a comprehensive and emerging discipline across natural sciences, social sciences and humanities. The major offers great potential for development and involves knowledge of plastic arts, art design, interactive design, computer software technology, graphics and image processing, and information and communication technology. This major’s aim is to cultivate students’ talents in artistic creation and digital product art design. As an application-oriented major, students also need to work effectively in a team and communicate clearly – abilities that coincide with innovation and entrepreneurship education concepts.

The state promotes innovation and entrepreneurship education in the field of higher education, and by placing the concept of ‘innovation’ before ‘entrepreneurship’, emphasises the direction of entrepreneurial behaviour in the digital era. Therefore, the cultivation of innovative, entrepreneurial talents can help achieve a high level of guidance, improve the level of entrepreneurship and promote industrial transformation and upgrading. The purpose of innovation and entrepreneurship education reform in the digital media art design specialty is to cultivate innovative quality and entrepreneurial ability. The mode is innovative entrepreneurship practice, based on professional ability, and the carrier is high-level professional talent training with comprehensive quality.

Firstly, in terms of professional talent training, the orientation of talent training objectives in colleges and universities should be closely related to social needs. There is a certain development of students’ innovative thinking in digital media art design through the professional talent training programme and curriculum structure. However, the focus is mainly on improving professional quality. Based on professional skills and the professional development of entrepreneurial quality, training is more for professional teachers. This often occurs in the teaching process spontaneously, lacking a systematic, comprehensive and long-term view.

The goal of cultivating innovative and entrepreneurial talents is not to require everyone to become an inventor and entrepreneur but to cultivate innovative talents and qualities that support social and economic transformation and development in the future. With the rapid development of information technology and the industrial economy, the change in social demands is great. The talent training goal of digital media art design should also keep pace with the times. To cultivate creativity and creative ability, we should pay attention to the development of student innovation and entrepreneurship. We should also break the previous solidified, talent training mode and rigid teaching management when designing professional curricula. The teaching system for each professional course should be reconstructed to achieve the goal of professional talent training. The cultivation of innovation ability should be regarded as the link between entrepreneurship education and professional design. Innovation and entrepreneurship should be systematically integrated into professional courses, and practical teaching concepts, innovation and entrepreneurship guidance should run organically through the entire professional education process.

The cultivation of innovative and entrepreneurial talents in the digital media art design major should not only cultivate students’ ability to generate new ideas, discover new elements and create new things, but also cultivate digital media cognitive ability, creative ability and processing ability, as well as the comprehensive quality of digital media security, ethics and property rights. Therefore, in terms of talent training mode, we should also abandon the previous single talent training mode of professional ability training and entrepreneurial ability training, as well as relying mainly on the school curriculum.

The core of professional talent training is the curriculum content. For the innovation and entrepreneurship education reform of interdisciplinary disciplines, with strong practicality and technology, such as digital media art and the high requirements for professional, innovative ability and creative thinking, in particular, it is necessary to constantly explore the curriculum content and practical experiences. The aim is to build a set of curriculum systems with creativity as the core, through a perfect and organic professional curriculum system, with the relevant content to strengthen the practical effect of innovation and entrepreneurship education. The training goal of the digital media specialty is to develop and design compound talents innovatively. Therefore, the curriculum system and teaching practice of this specialty must closely follow the ‘innovative’ and ‘compound’ elements of the digital media design industry, driving the development of the practical teaching system with projects, adjusting the focus of practical teaching with new theories, new viewpoints and new technologies, and strengthening the innovation and practicality of project-practice-driven curriculum reform teaching.

Innovation and entrepreneurship education need to operate a systematic policy system. Systematic promotion requires not only the principle of macro guidance at national level, but also the existence of diversified support systems at all levels. The purpose of cultivating the innovative and entrepreneurial talents of the digital media specialty is to develop the talents of students alongside creativity, strong professionalism and broad vision. We must focus on this goal both in talent training objectives and in the construction of a curriculum system. Subsequently, we should also make adjustments to the teaching management system and the teaching effect evaluation, striving to be consistent for the purposes of professional innovation and entrepreneurship talent training, as this is the external guarantee condition for the smooth operation of the talent training mode. For example, at teaching management level, the support for innovation and entrepreneurship is reflected in flexible teaching system support, rich teaching link arrangements and support, support for diversified learning approaches, credit certification system support and corresponding support for the teaching effect evaluation system.

Secondly, the innovation and entrepreneurship education of digital media art design require a more organic curriculum integration mechanism. Our study suggests that innovation and entrepreneurship education, as well as professional education, are relatively independent, a reality for most colleges and universities. Professional education often focuses on cultivating students’ professional knowledge and ability but concentrates less on entrepreneurial consciousness and the development of ability. Entrepreneurship education focuses on business and development but lacks the support of professional knowledge. To solve this problem, a curriculum integration mechanism is needed. Colleges and universities should make it clear that entrepreneurship education is not a supplement to professional education. Entrepreneurship education courses should be added to professional courses, and a multi-level ‘in class’ and ‘out of class’ curriculum integration mechanism should be promoted, based on the degree of correlation between specialty and entrepreneurship.

From the results of interviews with successful professionals from the world of digital media art design, the curriculum system and practical experience in school correlate strongly with students’ willingness to participate in innovation and entrepreneurship practice. The digital media art design major – given the nature of the course and teaching form – can be divided into four different areas: general basic professional courses, partial integration professional courses, full integration professional courses and second classroom business practice courses. Each of the four levels has its own emphasis, but they cannot be separated. General basic professional courses are often very theoretical, with few links to entrepreneurship courses and are often set up in the lower year groups. Such courses can integrate innovation and entrepreneurship practice cases, related to the course content in the teaching process. The purpose is to enhance students’ innovation and entrepreneurship awareness. The partial integration professional course combines innovation and entrepreneurship practice with course content and teaching objectives. The course requirements introduce practical projects in enterprises or markets to develop students’ professional knowledge and further understand the basic qualities related to entrepreneurship, e.g. project processes and market demand. A full integration of professional courses means that entrepreneurial practice content is completely embedded in professional courses, including enterprise practice courses and interdisciplinary comprehensive project practice courses. These courses are typically established in the senior year groups. By this time, students have relatively solid professional and technical skills, and a clear understanding of the process and significance of professional entrepreneurship. Now, the whole process of entrepreneurship and professional ability can be organically integrated into the course. Students may have some entrepreneurial ideas before graduation but can also test their professional ability in the process of entrepreneurial practice. The layered curriculum integration mechanism of combining specialty, layered teaching and strengthening capabilities in terms of practice within these professional courses plays a role in cultivating innovative and entrepreneurial talents, thus producing a positive effect of high popularity, strong professional relevance and the development of ability.

Thirdly, teachers are mainly responsible for implementing education and teaching, and the construction mechanism of the teacher team plays an indispensable role in the innovation and entrepreneurship practice teaching of the digital media art design major. In the interviews, we found that most of the teachers of innovation and entrepreneurship education in colleges and universities were counsellors, administrators and ideological and political teachers. Innovation and entrepreneurship education were based on general theory and education, often resulting in teachers’ promotion and progression in education, but moving them away from their professional background and direction. Although the teachers of the digital media art design major have rich professional knowledge and a solid professional foundation, they have only explored theory, knowledge and methods of innovation and entrepreneurship education in a limited way, and struggle to achieve a real integration of the two.

The education reform of the digital media art design specialty needs to expand to build a teaching team with solid, professional and entrepreneurial practice experience from multiple channels. We can encourage teachers to participate in enterprise and entrepreneurship practice and strengthen teachers’ innovation and entrepreneurship education ability. An alternative approach would be to employ enterprise teachers in schools to focus on the advantages of enterprise teachers’ practical experience, and to better implement the organic integration of innovation and entrepreneurship education, and professional curriculum content.

A more flexible teacher cooperation mechanism could be developed. In some courses within the digital media art design major, with a high integration of professional skills and entrepreneurial practice, ‘dual mentor’ teaching could be adopted with school teachers and out-of-school professionals, engaged in innovation and entrepreneurship education. ‘Dual mentors’ refer to innovation and entrepreneurship mentors and professional mentors. Innovation and entrepreneurship mentors refer to teachers in school and out-of-school professionals engaged in innovation and entrepreneurship education, mainly responsible for curriculum implementation and process support for innovation and entrepreneurship. The terms professional tutors refer to teachers or enterprise staff selected from the college, with practical experience and practical ability in industry, university and research. They encourage and guide students to participate in innovation and entrepreneurship practice by designing project teams.

In this way, students could benefit from two-way support in terms of professional innovation ability and entrepreneurial process.

Teachers should play a key role in the integration of innovation and entrepreneurship education with the specialty. The establishment and improvement of incentive and support policies are key in mobilising teachers’ enthusiasm and changing their understanding of educational reform. The school could encourage teachers, who contribute to the integration of professional education and entrepreneurship education through the incentive policies of professional title promotion, performance appraisal, training and further study. At the same time, schools should also provide or increase training platforms and practical opportunities to help teachers improve their ability, by developing external forces, such as government support, enterprise cooperation and social support. Teachers need to be supported to become the backbone and core of innovation and entrepreneurship education.

Fourthly, the school is the main body for the integration of professional and entrepreneurship education. The effective development of innovation and entrepreneurship education requires strong support and guarantees from the school regarding campus culture, organisation, practice sites, resource platforms and capital. The integration and promotion of students’ professional core competences and innovation, as well as entrepreneurship ability must use practice as the carrier, and practical activities need space, equipment and funds (Lin et al., 2020). The innovation and entrepreneurship education reform of professional education should actively strive for the support of university resources, such as the construction of ‘maker space’, college students’ entrepreneurship incubation base, entrepreneurship parks and other campus practice platforms, the improvement of project training equipment and environments, the increase of teacher training funds and the establishment of an effective innovation and entrepreneurship campus culture. The application process for public resources needs to be simplified and online and offline innovation, and entrepreneurship resource platforms should be developed inside and outside the school to help students exercise their professional ability more comprehensively in the practice process, stimulate their innovation and entrepreneurship potential and foster their enthusiasm for innovation and entrepreneurship.

Finally, the school-enterprise cooperation mechanism is also an indispensable element of the innovation and entrepreneurship capabilities of the digital media art design major, which can help in three areas, namely, curriculum integration, teacher team construction and resource support external to colleges and universities. Colleges and departments should actively establish an efficient, flexible and win-win cooperation mechanism with professional enterprises. Enterprise is the main body of project practice and the result of innovation and entrepreneurship activities. The innovation and entrepreneurship education practice of digital media art design in colleges and universities will eventually be tested by the market (Erasmia et al., 2020).

Digital media art design enterprises have more practical projects and a more practical environment than many other enterprises engaged in the real economy. Cooperation with such enterprises can help schools alleviate the internal pressure for training places, practice curriculum development and teacher team construction, facilitate practical opportunities and provide project funding support, among other aspects. Colleges and universities can cooperate with enterprises to develop courses, introduce advanced industry and enterprise standards, discuss talent training programmes and bring new technologies and specifications of enterprises into teaching content, based on the actual needs of enterprises. When teaching courses, we can carry out discussions with cooperating enterprises, select the appropriate resources for enterprise practice projects, integrate practice links into relevant courses, realise actual projects in the classroom and integrate theory teaching, basic skills learning and operation training. We need to ensure that the teaching content is up to date to carry out systematic, practical teaching using comprehensive professional knowledge. We want students to intuitively understand the social needs of professional-related directions and the operation of enterprises, accumulating innovation and entrepreneurship experience.

In terms of the construction mechanism of the teacher team, schools can make good use of the practice resources of cooperative enterprises to select key teachers or teachers of innovation and entrepreneurship courses, to participate in project training in enterprises. Teachers can understand the advanced management mode of enterprises and industries in terms of enterprise practice and can explain the specific needs of enterprises to professional individuals so as to better grasp the future direction of teaching design and its related content. Schools can also employ experts and professional ‘backbones’ from enterprises as part-time teachers to participate in professional education and teaching, innovation and entrepreneurship practice, graduation design guidance, entrepreneurship competition advice, internship teaching and textbook content compilation. This would help develop an innovation and entrepreneurship education and teaching team that integrates enterprise professionals, school professional teachers and innovation and entrepreneurship curriculum teachers. This kind of two-way talent interaction between enterprises and schools enables professional teaching to introduce current technology so that the design creativity of teachers and students more accurately reflects the market demand, ultimately improving students’ professional knowledge and skills (Zhang et al., 2012).

School-enterprise cooperation can also enrich the integration of entrepreneurship education and professional education in digital media art design. Based on the enterprise resources, schools can develop and build an off-campus practice platform, conducive to the organic integration of professional education and innovation, as well as entrepreneurship education. Once students have comprehensive professional skills, on-campus instructors can facilitate their participation in project practice or training in school-enterprise cooperation. Students participate in real enterprise projects in which they carry out practical training in innovation and entrepreneurship. They learn skills in the school system that they apply in enterprise to improve their ability in practice. Students understand the positioning and needs of the industry through their internship and learn about professional technology to enhance their professional value. Enterprises can cultivate and accept talented students, working together to achieve a win-win situation.

Cooperation between schools and enterprises requires a series of laws, regulations, systems, teacher teams and other factors. The effect of integration lies in resource sharing, mutual penetration and the formation of a mutually beneficial situation.

The major in digital media art design requires cooperation between schools and enterprises and can practically benefit both in terms of resources and results. Enterprises can benefit from a rich pool of creative ideas through cooperation with schools and can further promote achievements to help enterprises maintain the advantages of professional creativity and market competitiveness. Enterprises can also cultivate and reserve skilled professionals within the process of cooperation with the school. More importantly, the school and enterprise can establish a feedback mechanism for entrepreneurial activities, that is, both organisations can support beneficial entrepreneurial projects by providing resources, funds and projects. New school-enterprise cooperation relationships or investments may then come about after the successful completion of related entrepreneurial projects.

### Significance and Limitations

Innovation and entrepreneurship are future trends of national development and a way in which talents realise their self-worth. In the digital media industry, innovation and practice ability are key factors for entrepreneurship success, and the focus of digital media professional education. With the advent of the information age and the continuous development of digital technology, students majoring in digital media art and design have more development opportunities and face greater demands. The impact of COVID-19 on the global business economy has revealed that the future social economy is more inclined to develop the digital economy. Professional and technological talents are important for future economic and social development in the digital media art design industry. The purpose of innovation and entrepreneurship education reform of the digital media art design specialty is to integrate the cultivation of innovative quality and entrepreneurial ability. Innovation and practical ability are the key factors for entrepreneurship success and constitute the main focus of digital media education. To make innovation and entrepreneurship education more professional and to cultivate high-quality talent, teachers of the digital media design major in colleges and universities should pay attention to students’ artistic development and technological innovation and should integrate innovation and entrepreneurship education with professional education.

Through the investigation of the two dimensions of ‘innovation’ and ‘entrepreneurship’ and the two perspectives of ‘implementers’ and ‘parties’, this study has obtained more comprehensive, original research materials than previous studies, and uses the grounded theory research method to make a level-by-level coding analysis and to research the universality of the digital media art design specialty in the development of innovation and entrepreneurship education. We presented an education reform path model for innovation and entrepreneurship in the digital media art design major with five main categories of resource support mechanisms and the school-enterprise cooperation mechanism. We summarised methods, paths and the specific content for the organic integration of innovation and entrepreneurship education in digital media art design. By embedding innovation and entrepreneurship education into talent training programmes and courses, we can promote the establishment and improvement of curriculum integration mechanisms, as well as the creation of a team of innovation and entrepreneurship teachers. At the same time, with the support of resource support mechanisms and the school-enterprise cooperation mechanism, five levels of integration, cooperation and mutual support can stimulate students’ entrepreneurial consciousness and spirit. Students’ enthusiasm for acquiring professional knowledge may be enhanced and their innovation and entrepreneurial thinking and ability improved, providing important development space for more students. The research findings provide some direction for teaching reform and the professional construction of the digital media art design major. This is important to ensure responsiveness to the transformation and upgrading of economic and social development for the higher-level needs of digital media art design professionals. Furthermore, the methods and paths may have value for other similar majors in which innovation and entrepreneurship education need to be more professional and effective.

The limitations of this study are reflected in two aspects. Firstly, although the selection of the research subjects contains the two dimensions of ‘innovation’ and ‘entrepreneurship’ and the two perspectives of ‘implementers’ and ‘parties’, the number of participants was relatively small. Secondly, innovation and entrepreneurship education reform and the practical element of the digital media art design major also involve policy managers and students, who could have been included in the project. The next steps of the research will extend the aforementioned concepts to broaden the scope of the research subjects, verify theoretical saturation and further develop the ideas and direction of innovation and entrepreneurship education reform within the digital media art design major.

## Data Availability Statement

The raw data supporting the conclusions of this article will be made available by the authors, without undue reservation.

## Ethics Statement

This study did not involve clinical medical research and privacy issues of interviewees, and therefore ethical approval was not required from our university. No informed consent was required because the contents of the interviews were anonymized.

## Author Contributions

WHY: conceptualisation, methodology, investigation, formal analysis, visualisation, and writing. TJ: investigation, formal analysis, validation, and editing. The collection of research data in this study is divided into two directions. Yu is mainly responsible for industry and related companies, and TJ is mainly focus on interviews with respondents who come from universities and education institution. They worked together to complete the data analysis and induction work from open coding to axial coding. The process from axial coding to selective coding was mainly completed by Yu. All authors contributed to the article and approved the submitted version.

## Conflict of Interest

The authors declare that they have no known competing financial interests or personal relationships that could have influenced the work reported in this paper.

## Publisher’s Note

All claims expressed in this article are solely those of the authors and do not necessarily represent those of their affiliated organizations, or those of the publisher, the editors and the reviewers. Any product that may be evaluated in this article, or claim that may be made by its manufacturer, is not guaranteed or endorsed by the publisher.

## Appendix: Questionnaire of the Semi-Structured Interviews of the Research

PART A, for leaders of the major of digital media art design courses in colleges and universities:

Q1. What do you think are the necessary core competencies of innovative and entrepreneurial students majoring in digital media art design?

Q2. What do you think are the convergence points between digital media art and design, professional talent education, and innovation and entrepreneurship education?

Q3. What do you think are the advantages of carrying out innovation and entrepreneurship education reform within the digital media art design major?

Q4. In your opinion, what are the common problems and difficulties in developing innovation and entrepreneurship education in digital media art design?

Q5. What support measures does your school have for innovation and entrepreneurship education reform of the digital media art design major?

6. What specific methods and successful experiences does your school have in relation to innovation and entrepreneurship education reform of the digital media art design major?

Q7. What do you think are the main factors influencing the combination of the digital media art design major and innovation and entrepreneurship education?

Q8. In your opinion, what are the directions and methods for innovation and entrepreneurship education reform of the digital media art design major? Which are the most important?

PART B, for successful entrepreneurs who have majored in digital media art design:

Q1. What do you think are the necessary core competencies of innovative and entrepreneurial students majoring in digital media art design?

Q2. What do you think are the convergence points between digital media art and design, professional talent education and innovation and entrepreneurship education?

Q3. What do you think are the advantages of carrying out innovation and entrepreneurship education reform of the digital media art design major?

Q4. What are the main problems and difficulties you encountered in the combination of innovation and entrepreneurship education, and professional education during your university study?

Q5. Which methods of innovation and entrepreneurship education in your university are most helpful to your entrepreneurship?

Q6. How do you rate the current demand for innovative and entrepreneurial talent in the digital media art design industry and enterprises?

Q7. What do you think are the main factors influencing the combination of the digital media art design major and innovation and entrepreneurship education?

Q8. In what aspects do you think enterprises can cooperate with colleges and universities to cultivate professional, innovative and entrepreneurial talents jointly?
