# Supplementary material for: Research on the Direction of Innovation and Entrepreneurship Education Reform Within the Digital Media Art Design Major in the Digital Economy
Source: Front Psychol. 2021 Oct 15;12:719754. doi: 10.3389/fpsyg.2021.719754 (PMC8554304; doi:10.3389/fpsyg.2021.719754)
Supplement: Supplementary file 1 [file Data_Sheet_1.docx]

**Appendix**

**Questionnaire of the Semi-Structured Interviews of the Research**

**PART A. For leaders of the major of digital media art design courses in colleges and universities:**

Q1. What do you think are the necessary core competencies of innovative and entrepreneurial students majoring in digital media art design?

Q2. What do you think are the convergence points between digital media art and design, professional talent education, and innovation and entrepreneurship education?

Q3. What do you think are the advantages of carrying out innovation and entrepreneurship education reform within the digital media art design major?

Q4. In your opinion, what are the common problems and difficulties in developing innovation and entrepreneurship education in digital media art design?

Q5. What support measures does your school have for innovation and entrepreneurship education reform of the digital media art design major?

Q6 .What specific methods and successful experiences does your school have in relation to innovation and entrepreneurship education reform of the digital media art design major?

Q7. What do you think are the main factors influencing the combination of the digital media art design major and innovation and entrepreneurship education?

Q8. In your opinion, what are the directions and methods for innovation and entrepreneurship education reform of the digital media art design major? Which are the most important?

**PART B. For successful entrepreneurs who have majored in digital media art design:**

Q1. What do you think are the necessary core competencies of innovative and entrepreneurial students majoring in digital media art design?

Q2. What do you think are the convergence points between digital media art and design, professional talent education and innovation and entrepreneurship education?

Q3. What do you think are the advantages of carrying out innovation and entrepreneurship education reform of the digital media art design major?

Q4. What are the main problems and difficulties you encountered in the combination of innovation and entrepreneurship education, and professional education during your university study?

Q5. Which methods of innovation and entrepreneurship education in your university are most helpful to your entrepreneurship?

Q6. How do you rate the current demand for innovative and entrepreneurial talent in the digital media art design industry and enterprises?

Q7. What do you think are the main factors influencing the combination of the digital media art design major and innovation and entrepreneurship education?

Q8. In what aspects do you think enterprises can cooperate with colleges and universities to cultivate professional, innovative and entrepreneurial talents jointly?
